# Clinical approach to the knotting diagnostic quadripolar catheter during zero‐fluoro electrophysiological study

**DOI:** 10.1002/ccr3.2123

**Published:** 2019-03-29

**Authors:** Vern Hsen Tan, Chi Keong Ching, Kelvin Cheok Keng Wong

**Affiliations:** ^1^ Cardiology Department Changi General Hospital Singapore City Singapore; ^2^ Cardiology Department National Heart Center Singapore Singapore City Singapore

**Keywords:** electrophysiology procedure, knotting of catheter, unravel

## Abstract

We report a case of electrophysiology catheter knotting when inserted without fluoroscopic guidance followed by technique to unravel the knot via ipsilateral femoral vein. We advocate caution when manipulating catheters using minimal/zero fluoroscopic technique and having a low threshold to screen under fluoroscopy when encountering difficulties during catheter insertion.

## 1 CASE REPORT

A 74‐year‐old man who presented with symptomatic adenosine‐sensitive supraventricular tachycardia underwent electrophysiology study and ablation.

The procedure was initially conducted using three‐dimension electroanatomic mapping system (Ensite Precision™ Cardiac Mapping System, St Jude Medical Inc, St Paul, MN, USA) without the use of fluoroscopy.

Three catheters were used for the electrophysiology study via right femoral vein [Livewire 6 French (F) decapolar catheter (St Jude Medical Inc, St Paul, MN, USA) was placed at coronary sinus, Avail Josephson 6F quadripolar catheter (Johnson & Johnson Medical Inc, New Brunswick, NJ, USA) was placed at right ventricular apex and CRD‐2 6F quadripolar catheter (St Jude Medical Inc, St Paul, MN, USA) was placed at His].

**Figure 1:**
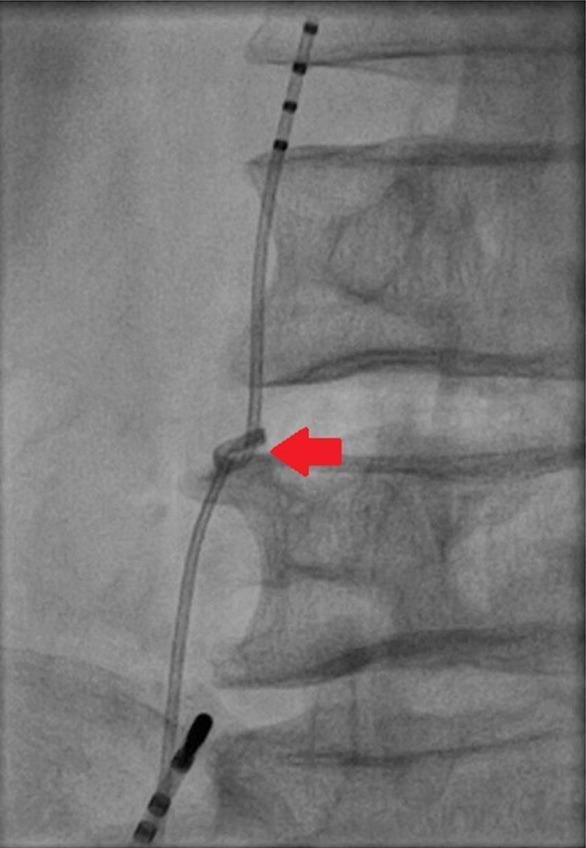
Knotting (measuring 6.7 mm × 4.7 mm) of quadripolar catheter

After completing electrophysiology study, we were unable to withdraw the quadripolar catheter. On fluoroscopy, the quadripolar catheter was found to be knotted. The knot measured 6.7 mm by 4.7 mm (Figure 1).

We considered different options to remove the knotted catheter. One option was to remove the knotted catheter by right femoral vein cut down at the puncture site. Another option was to snare the knotted catheter by gaining assess on the contralateral femoral vein using a larger sheath (at least 21F internal diameter). Both methods may potentially cause vascular damage and preclude subsequent ablation procedure. We decided to unravel the knot using a percutaneous approach. To achieve this, we needed to get through the center of the knot and exert forces in opposite direction to unravel the knot.

**Figure 2:**
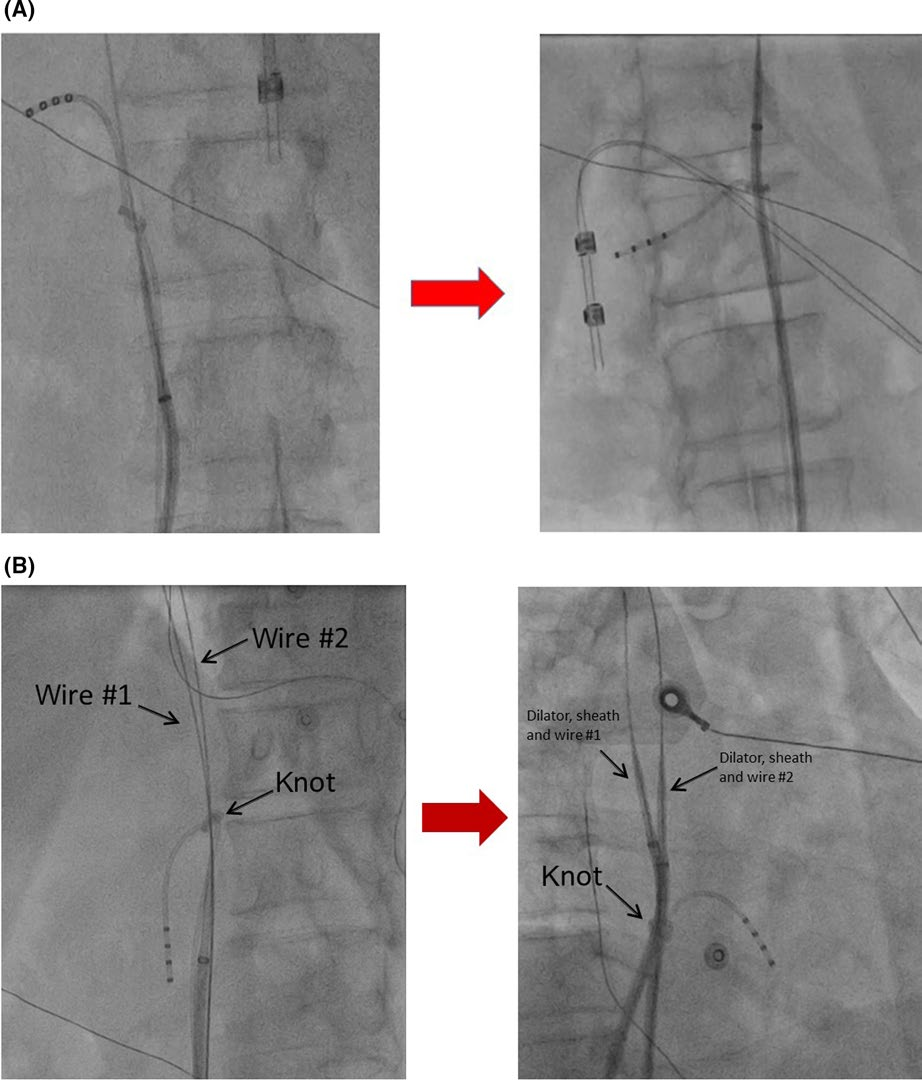
A, Wire crossed through the knot (left figure) followed by SRO sheath and dilator (right figure) while maintaining traction on the catheter (push and pull technique) in order to “open up” the knot. B, Using the retained wire (wire #1) technique, another wire (wire #2) was crossed the knot (left figure). This was followed by advancement of both dilator and sheath across the knot (right figure)

A long sheath (SRO, 8.5F) together with dilator and stiff guide wire (0.025″ in diameter and 180 cm in length) was inserted via the right femoral vein. The guide wire was then maneuvered through the knot followed by the dilator and sheath while maintaining traction on the quadripolar catheter (Figure 2A) under fluoroscopy guidance. Next, using the retained wire technique (after retracting both the long sheath and dilator below the knot), another SRO long sheath and wire was used to cross the knot. The advancement of both long sheaths with dilator across the knot further “opened it up” (Figure 2B). Next, a deflectable ablation catheter was inserted through one of the long sheaths to exert traction on one side of the knot by fully flexing the ablation catheter. For the other long sheath, the tip of the sheath was caught against the other side of the knot (dilator was removed but stiff wire was retained across the knot) providing counter‐traction. Using traction and counter‐traction method, we attempted to unravel the knot. However, it was unsuccessful as the deflected ablation catheter alone did not provide sufficient traction to unravel the knot.

**Figure 3:**
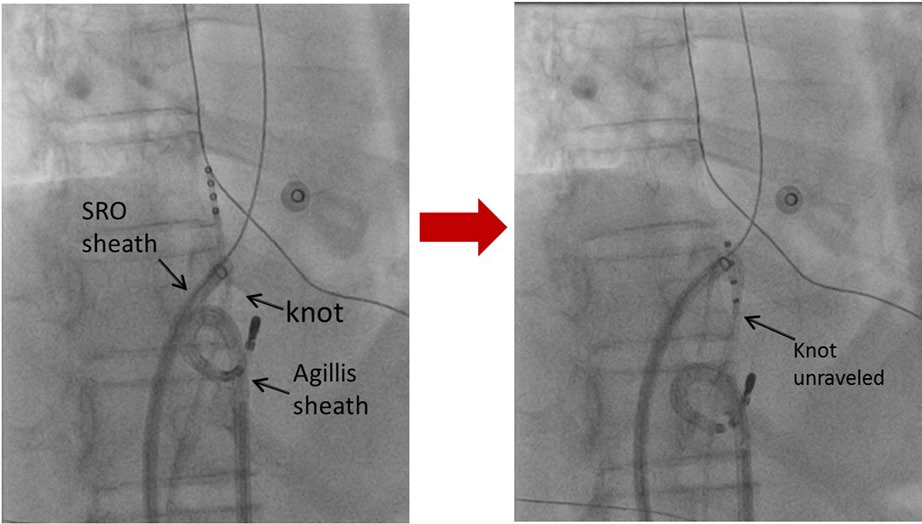
Using traction and counter‐traction technique, the knot was unraveled (right figure) by using Agilis™ sheath together with SRO sheath (left figure; Video S1)

In order to improve traction, the SRO sheath was replaced with an Agilis™ (St Jude Medical Inc, St Paul, MN, USA) steerable sheath (8.5F/91 cm). Using an ablation catheter through Agilis™ sheath followed by full deflection of Agilis™ sheath, we were able to exert enough traction while maintaining counter‐traction with the other SRO sheath tip to unravel the knot (Figure 3).

We were then able to proceed to perform a successful ablation of the concealed right anterolateral accessory pathway. Eventually, the additional fluoroscopy time and radiation dose required to guide the unraveling of the knotted catheter was estimated to be about 30 minutes and 80 000 mGy/cm^2^, respectively.

## 2 DISCUSSION

Increasingly, catheter ablations can be performed with minimal or even zero fluoroscopy, leading to significant reduction in radiation dose as well as fluoroscopy time.^1^, ^2^ In addition, studies have shown that the minimal fluoroscopic technique was safe and did not compromise acute success rates.^1^, ^2^

Knotting of intravascular catheter was first reported in 1954.^3^ Since then, many more cases were published,^4^ with majority of the cases involving pulmonary artery catheters which were inserted without the use of fluoroscopy.^4^

The mechanism of catheter knotting in our case was possibly due to continued “blind” advancement and torqueing of catheter despite resistance, leading to the formation of a loop and subsequently a knot without the operator's knowledge. The knot then became “tightened” when the catheter was withdrawn against the sheath. It is prudent to stop advancing the catheter when resistance is met or when the tip of the catheter appears “stuck” and not advancing as expected, but to retract the catheter before re‐advancing. There should also be a low threshold to use fluoroscopy to screen for the position of catheter when encountering resistance during catheter advancement.

Generally, there are two approaches to deal with catheter knotting. The percutaneous (nonsurgical) approach is preferable while the surgical approach is reserved for large or multiple loop knots. Using the nonsurgical approach, one can attempt direct removal of the knotted catheter (cut down technique or upsizing to bigger introducer sheath) or use specific tools (retrieval basket, loop snares or steerable catheter) to unravel the knot before removal.^5^, ^6^, ^7^, ^8^

In our case, we improvised by using readily available sheaths and catheters (SRO sheath and Agilis™ steerable sheath) in the electrophysiology catheter laboratory to unravel the knot. The use of Agilis™ steerable sheath was necessary to improve traction together with the SRO sheath providing counter‐traction, to finally unravel the knot.

Plasek et al^9^ recently published and described a technique of unraveling knotted decapolar catheter using Agilis™ steerable sheath. The author approached the case with Agilis™ steerable sheath inserted via right internal jugular vein. The Agilis™ sheath could easily cross the large knot. Using traction (pulling the Agilis™ sheath from the superior approach) and counter‐traction (pulling the decapolar catheter inferiorly in the opposite direction) technique, the knot was successfully unraveled.

The similarity between Plasek et al's^9^ case and our case was the use of Agilis™ steerable sheath. The Agilis™ steerable sheath provides stability, versatility with improved steering capability during knot unraveling process. The difference between Plasek et al's^9^ case and our case was that the loop of the knot was much smaller in our case, thus posing a significant challenge to the unraveling process. In addition, we were able to perform traction and counter‐traction by approaching the knot using the same femoral vein access, using two sets of sheaths described above.

## 3 CONCLUSIONS

Increasingly, electrophysiological studies are being performed with minimal or even zero fluoroscopic guidance. We report a case of catheter knotting which may be a potential complication when inserted without fluoroscopic guidance. We report a technique to unravel the knot using two sheaths inserted via the ipsilateral femoral vein. We also advocate caution when manipulating catheters when using the minimal/zero fluoroscopic technique and having a low threshold to screen under fluoroscopy when encountering difficulties during catheter insertion.

## CONFLICT OF INTEREST

None declared.

## AUTHOR CONTRIBUTION

VHT: Contributed to the conception and design of the work, drafting, revising the work, and made the final approval as well as accountable for all aspects of the work; CKC: Contributed to the revision of the manuscript and accountable for all aspects of the work; KCKW: Contributed to the conception and design of the work, revising the work, and made the final approval as well as accountable for all aspects of the work.
